# Redescription and new record of *Paracapillaria* (*Ophidiocapillaria*) *najae* (Nematoda: Trichuroidea) in the monocled cobra *Naja kaouthia* from central Thailand: morphological and molecular insights

**DOI:** 10.1017/S0031182023000707

**Published:** 2023-09

**Authors:** Vachirapong Charoennitiwat, Kittipong Chaisiri, Sumate Ampawong, Panithi Laoungbua, Lawan Chanhome, Taksa Vasaruchapong, Tanapong Tawan, Urusa Thaenkham, Napat Ratnarathorn

**Affiliations:** 1 Department of Helminthology, Faculty of Tropical Medicine, Mahidol University, Bangkok, Thailand; 2 Department of Tropical Pathology, Faculty of Tropical Medicine, Mahidol University, Bangkok, Thailand; 3 Snake Farm, Queen Saovabha Memorial Institute, The Thai Red Cross Society, Bangkok, Thailand; 4 Animal Systematics and Molecular Ecology Laboratory, and Applied Animal Science Laboratory, Department of Biology, Faculty of Science, Mahidol University, Bangkok, Thailand

**Keywords:** gastrointestinal helminth, monocled cobra, *Naja kaouthia*, *Paracapillaria* (*Ophidiocapillaria*) *najae*, snake parasite

## Abstract

The parasitic nematode *Paracapillaria* (*Ophidiocapillaria*) *najae* De, 1998, found in the Indian cobra *Naja naja* is redescribed and re-illustrated in the present study. The monocled cobra *Naja kaouthia* was discovered to be a new host for this parasite in central Thailand. A comprehensive description extending the morphological and molecular characteristics of the parasites is provided to aid species recognition in future studies. The morphometric characters of 41 parasites collected from 5 cobra specimens are compared with those described in the original studies. Phylogenetic analyses using mitochondrial cytochrome c oxidase subunit 1 and nuclear 18S ribosomal RNA genes were performed to provide novel information on the systematics of *P. najae*. Similar characteristics were observed in the examined nematode samples, despite being found in different hosts, confirming their identity as *P. najae*. The molecular genetic results support the species status of *P. najae*, indicating *P. najae* is well defined and separated from other related nematode species in the family Capillariidae. Morphological descriptions, genetic sequences, evolutionary relationships among capillariids and new host and distribution records of *P. najae* are discussed. *Paracapillaria najae* specimens found in the Thai cobra had some morphological variation, and sexual size dimorphism was also indicated. *Paracapillaria najae* was found to infect various cobra host species and appeared to be common throughout the Oriental regions, consistent with its hosts' distribution.

## Graphical abstract

**Figure:**
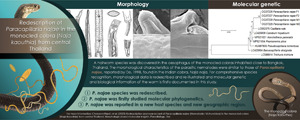

## Introduction

*Paracapillaria* Mendonça, 1963 is a genus of nematodes belonging to the family Capillariidae Railliet, 1915, whose members infect the digestive system of freshwater and marine fishes, amphibians, reptiles, birds, mammals and humans (Moravec, 1986; Moravec and Justine, 2020). This genus is divided into 3 subgenera: *Paracapillaria* Mendonça, 1963 (in fishes and amphibians), *Ophidiocapillaria* Moravec, 2001 (reptiles) and *Crossicapillaria* Moravec, 2001 (birds and mammals) (Moravec, 1986; Moravec and Justine, 2020). Notably, one of the species within the genus, *Paracapillaria* (*Crossicapillaria*) *philippinensis* (Chitwood *et al.*, 1968), has the potential to be zoonotic, causing intestinal capillariasis in humans (Chitwood *et al*., 1968; El-Karaksy *et al*., 2004; Saichua *et al*., 2008).

Although *Paracapillaria* has been extensively studied, certain aspects of this genus, such as its infection in wildlife species, particularly snakes of the subgenus *Ophidiocapillaria*, remain poorly understood. Over the past century, at least 12 *Paracapillaria* species, found in various snake hosts, have been described (Teixeira de Freitas and Lent, 1934; Skryabin *et al*., 1957; Pence, 1970; Wang, 1982; Biserkov *et al*., 1985; Moravec, 1986; Moravec and Gibson, 1986; De, 1998) The subgenus *Ophidiocapillaria*, as established by Moravec (1986), is of particular interest. However, the identification of these closely related species has primarily relied on morphological data, with limited molecular data and often a restricted number of host and parasite samples. These limitations can pose challenges in recognizing *Ophidiocapillaria* species, especially when they are discovered in new host species or geographical areas. Furthermore, the morphometric characteristics of the parasites may not align with previously described morphology due to the small sample sizes. It is important to note that morphology-based identification mainly applied to the adult stage of the parasites and may not provide comprehensive genetic information. Therefore, relying solely on morphology may not be sufficient for the identification of *Ophidiocapillaria* species.

Between 2000 and 2022, authors' parasitic surveys led to the recognition of capillariid worms collected from the monocled cobras (*Naja kaouthia*) in central parts of Thailand. Other studies have reported the presence of the worms in the same snake host and locality, but specific identification has been challenging, resulting in taxonomic determination only at the level of *Capillaria* sp. (Chaiyabutr and Chanhome, 2002; Vasaruchapong *et al*., 2017). These parasites were predominantly found in the oesophagus of the cobras and displayed morphological similarities to *Paracapillaria* (*Ophidiocapillaria*) *najae*. This species was initially described by De (1998) based on specimens from the Indian cobra *Naja naja* in West Bengal, India. However, the measured characteristics of the parasite specimens, such as body length and width, significantly deviated from the recorded morphological ranges. There were also morphological variations and differences compared to the original study by De (1998).

Recently, phylogenetic and microstructure imaging have proven invaluable in differentiating parasite species and detecting minor morphological variations (Eisenback, 1986; Mattiucci and Nascetti, 2008; Cutillas *et al*., 2009). Traditionally, studies on paracapillariid species infecting snakes relied solely on taxonomic methods, neglecting the collection of genetic data for species within this subgenus. Regarding *P.* (*O.*) *najae*, De's (1998) report provided comprehensive morphological data. However, the reliance on a single host specimen, combined with the absence of genetic data and microscopic imaging, resulted in misunderstandings related to morphology and measurements, leading to ambiguous species identification.

The objective of this current study was to offer a more comprehensive description of *P. najae* specimens found in a new host, the monocled cobra (*N. kaouthia*), in central Thailand. This involved using scanning electron microscopy to examine the morphological and morphometric characteristics and comparing them with those of *P. najae*, as initially described by De (1998). In addition to providing extensive morphological descriptions and illustrations of *P. najae*, the present study aimed to facilitate more accurate species identification for future research. Furthermore, the molecular characterization of *P. najae* was conducted by employing sequences of mitochondrial cytochrome c oxidase subunit 1 (*COI*) and small subunit nuclear ribosomal RNA (18S rRNA) genes. This helped determine the phylogenetic position of *P. najae* among capillariid worms and provided a reference for future taxonomic identification. By integrating molecular and morphological data, the present study aimed to overcome the limitations of previous methods and enhance the understanding of this genus of parasites.

## Materials and methods

Five wild monocled cobra carcasses were donated by the snake farm at Queen Saovabha Memorial Institute of the Thai Red Cross Society in Thailand. Each snake was dissected to examine parasites in the oesophagus following the guidelines described by Toland and Dehne (1960). The organ was placed in a Petri dish quarter-filled with 0.85% normal saline before dissection, and it was examined under a stereo-microscope (Olympus, SZ30 and SZ51). The parasites were observed and isolated using micro-dissecting needles, and the paracapillariids were counted and placed in a small Petri dish filled with 0.85% normal saline. The parasites were then preserved in 70% ethanol at −20 °C until use. Some complete specimens were also preserved in glutaraldehyde for scanning electron microscopic imaging.

For the morphological studies, adult male and female worms were obtained from each of the 5 cobra hosts, resulting in the collection of a total of 21 males and 20 females. These parasites were randomly selected and examined under an inverted microscope (Zeiss, Primovert) equipped with a Zeiss Axiocam (ZEN2 blue edition software) to collect morphological information. Drawings were made under a light microscope with a camera lucida (Olympus, Thailand, Leitz) to illustrate the microscopic appearances of selected morphological characteristics of males and females. The measurement of these characteristics followed the methods described by Biserkov *et al*. (1994) and De (1998). To compare character differences between males and females that were not clarified in De's (1998) study, the Mann–Whitney *U* test was employed. All measurements were recorded in micrometres (μm).

To examine the *P. najae* worms using a scanning electron microscope (SEM), 3 male and 3 female specimens were preserved in 2.5% glutaraldehyde in 0.1 M sucrose phosphate buffer (SPB). The specimens underwent secondary fixation with 1% osmium tetroxide in 0.1 M SPB, dehydrated with ethanol, dried in a critical point drying device (CPD300 auto, Leica, Wetzlar, Germany), coated with a coat-sputter (Q150R PLUS, Quorum, East Sussex, England) and examined under an SEM (JSM-6610LV, JEOL, Tokyo, Japan).

For DNA extraction, 2 male and 2 female specimens of *P. najae* were selected. Each specimen was homogenized and processed using the DNeasy Blood and Tissue Kit (Qiagen, Germany) according to the manufacturer's instructions. The extracted genomic DNA was eluted with 30 μL of nuclease-free water and quantified using spectrophotometry.

A partial sequence of *COI* gene was amplified from the selected samples. This gene locus has been previously demonstrated to be useful for resolving genetic divergence within nematode species (Chan *et al*., 2020). Additionally, a nuclear gene, 18S rRNA, was amplified to support the results of the mitochondrial DNA analysis (Tokiwa *et al*., 2012; Eamsobhana *et al*., 2015; Chan *et al*., 2020). The following primers were used: COI_Paracap_F 5′-AGTRTTTGGTCCTTTRGG-3′ and COI_Paracap_R 5′- GAWGCAT TAGAAAGAGA-3′ for the *COI* gene, and 1096F 5′-GGTAATTCTGGAGCTAATAC-3′ and 1916R 5′-GGTAATTCTGGAGCTAATAC-3′ for the 18S rRNA gene. The amplifications were carried out using a T100 thermocycler (Bio-Rad, California, USA). The polymerase chain reaction (PCR) reaction mixture consisted of a final volume of 30 μL containing 15 μL of 2× i-Taq master mix (Biotechnology, Gyeonggi, South Korea), 10 μm of each primer and 1 ng μL^−1^ of DNA. For the *COI* primers, the thermocycling profile was as follows: an initial denaturation step at 95 °C for 5 min, followed by 30 cycles of 95 °C for 30 s, 52 °C for 1 min and 72 °C for 45 s. The reaction concluded with a final extension step at 72 °C for 5 min. Regarding the 18S rRNA primers, the thermocycling profile consisted of an initial denaturation at 94 °C for 5 min, followed by 5 cycles of 94 °C for 30 s, 45 °C for 30 s and 72 °C for 70 s, and 35 cycles of 94 °C for 30 s, 54 °C for 30 s and 72 °C for 70 s. The reaction concluded with a final extension step at 72 °C for 5 min, as described by Holterman *et al*. (2006). To visualize the PCR amplicons, a 1% agarose gel stained with SYBR safe (Thermo Fisher Scientific, Waltham, USA) was used. Subsequently, the PCR products were subjected to sequencing using Barcode Taq sequencing, which does not require primer walking (Celemics, Seoul, South Korea). The nucleotide sequences of the parasites obtained from this study were deposited in the NCBI database, and the corresponding GenBank accession numbers can be found in Figs 3 and 4.

The partial sequences of the 2 target genes were verified through manual inspection of electropherograms. The complementary strands were compared and adjusted using BioEdit version 7.2.5. (Hall, 1999). To analyse the phylogenetic relationships, a phylogenetic tree was constructed using the obtained sequences and additional sequences from parasites of Capillariidae, Trichuridae and Trichinellidae retrieved as outgroups from GenBank. The alignment of the data matrix was performed using ClustalX 2.1 (Hall, 1999; Thompson *et al*., 2003), and the aligned sequences were visually analysed using BioEdit. The aligned sequences were verified before conducting the phylogenetic analysis using maximum likelihood (ML) in MEGA-X. The best-fit nucleotide substitution model was determined, and the analysis was performed with 1000 bootstrap replicates (Tamura *et al*., 2013). A bootstrap value of over 70% is generally considered a strong level of support (Hillis and Bull, 1993). The phylogenetic trees were constructed using the ML method, with the Kimura 2-parameter (K) model with a gamma distribution for 18S rRNA and the Tamura 3-parameter (T92) model with a gamma distribution and evolutionarily invariable sites for *COI*.

## Results

### Family: Capillariidae Railliet, 1915 Genus: *Paracapillaria* Mendonça, 1963 Subgenus: *Ophidiocapillaria* Moravec, 2001 Species: *Paracapillaria* (*Ophidiocapillaria*) *najae* De, 1998 (Table 1, Figs 1 and 2)

### Host

*Naja kaouthia* Lesson, 1831.

**Table 1.** Comparison of the *Paracapillaria* (*Ophidiocapillaria*) *najae* specimens between De (1998) and present study

	De (1998)	Present study	
Host species	*Naja naja*	*Naja kaouthia*	
Locality	India	Thailand	
Male	*n* = 12	*n* = 21	
		Ranges	Average ± s.d.
Body length	16 840–24 970	11 579–25 592	19 069 ± 3902
Maximum width	85–113	56–123	93 ± 17
Nerve ring from anterior end	42–60	44–62	51 ± 6
Muscular oesophagus	193–266	210–374	287 ± 50
Stichosome	5420–8120	4635–9016	6630 ± 1091
Entire oesophagus	5660–8380	4855–9344	6916 ± 1116
Length of posterior part	–	6724–18 085	12 381 ± 3158
Ratio posterior: anterior ends	–	1.2–2.5	1.8 ± 0.4
Tail	17–24	14–38	24 ± 6
Spicule length	1800–2260	1437–2715	2032 ± 327
Ratio whole body:spicule length	–	7.9–11.4	9.4 ± 1.1
Female	*n* = 20	*n* = 20	
		Ranges	Average ± s.d.
Body length	16 060–22 820	16 532–31 485	22 518 ± 4465
Maximum width	113–134	101–180	133 ± 21
Nerve ring from anterior end	42–59	42–66	50 ± 8
Muscular oesophagus	255–321	148–436	305 ± 71
Stichosome	4850–6870	5719–8581	6895 ± 749
Entire oesophagus	5100–7160	5868–9249	7200 ± 771
Length of posterior part	–	9722–23 172	15 168 ± 3961
Ratio posterior:anterior ends	–	1.4–3.0	2.1 ± 0.5
Tail	8–19	12–25	20 ± 4
Vulva from oesophagus	38–104	46–107	68 ± 19
Egg length	57–71	60–71	66 ± 3
Egg width	23–32	28–34	31 ± 2
Number of eggs	–	127–545	285 ± 110
Vulval lips	–	Not elevated	

The micrometre (μm) is a unit of measurement.

**Figure 1.**
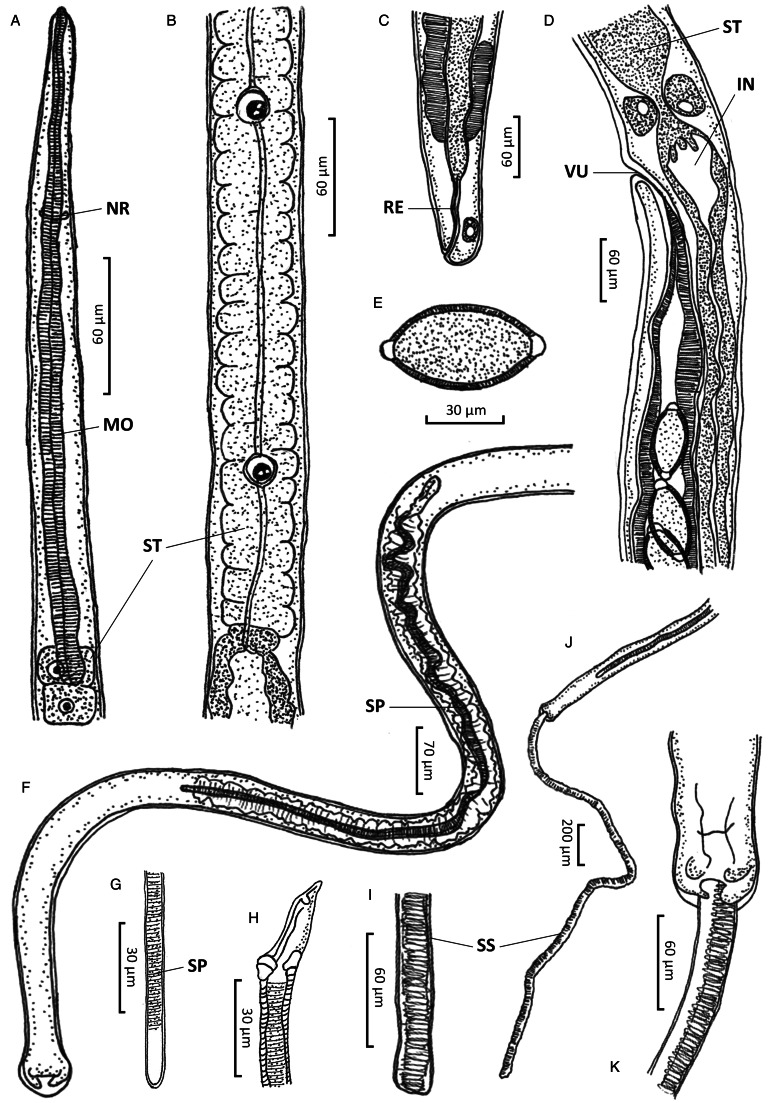
*Paracapillaria* (*Ophidiocapillaria*) *najae*. (A) Anterior end of male (lateral view). (B) Stichosome region of male. (C) Posterior end of female (lateral view). (D) Oesophago-intestinal junction of female (lateral view). (E) Fully developed egg. (F) Posterior part of male (ventral view). (G) Distal part of spicule (lateral view). (H) Proximal part of spicule (lateral view). (I) Spicular sheath (lateral view). (J) Posterior end of male (ventral view). (K) Posterior end of male (enlarged ventral view). IN, intestine; MO, muscular oesophagus; NR, nerve ring; RE, rectum; SP, spicule; SS, spicular sheath; ST, stichosome; VU, vulva.

**Figure 2.**
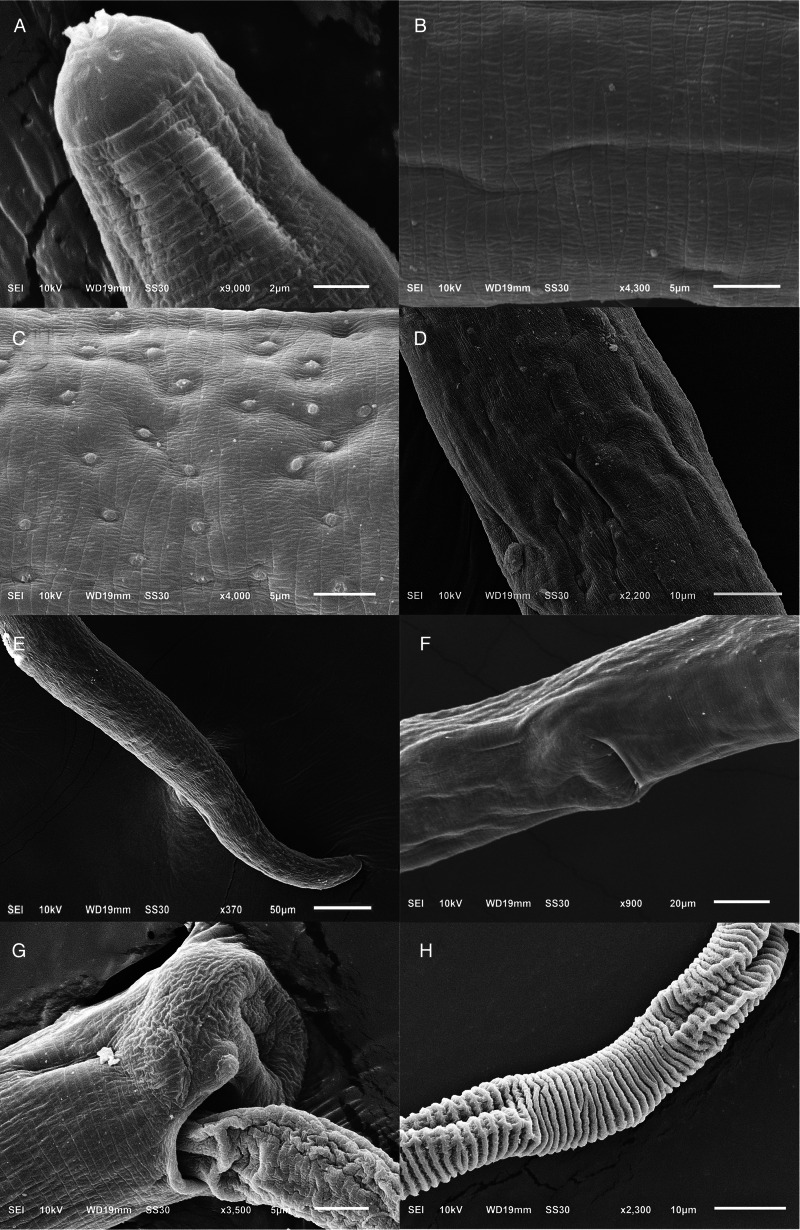
Scanning electron micrograph of *Paracapillaria* (*Ophidiocapillaria*) *najae*. (A) Anterior region of male (dorsal view). (B) Middle body with transverse striations (ventral view). (C) Middle body with button-like bacillary cells (dorsal view). (D) Middle body with bacillary band (dorsal view). (E) Posterior part of female (lateral view). (F) Middle region with dorso-lateral bacillary band and vulva opening (latero-ventral view). (G) Posterior end of male (lateral view). (H) Spicular sheath without spines.

### Collection date

10 November 2020 to 2 February 2022.

### Site of infection

Oesophagus

### New locality

Suburbs of Bangkok (e.g. Don Mueang and Prawet districts) and adjacent provinces, including Samut Prakan, Pathum Thani, Nonthaburi and Nakhon Pathom, in Thailand. Coordinates were not recorded.

### Redescription

Medium-sized filiform nematodes; body long and slender with a finely transversely striated cuticle (Fig. 2A and B). A bacillary band with button-like bacillary cells present dorso-laterally (Fig. 2C), extending between 2/5 of body length and cloaca (Fig. 2C–F). Males shorter and thinner than gravid females. Head rounded; mouth elevated (Figs 1A and 2A). Oesophagus with muscular part followed by stichosome; stichosome consisting of single row of 31–44 stichocytes; anterior stichocytes short; middle and posterior stichocytes long and subannulated with large nuclei (Fig. 1B). Nerve ring encircling muscular oesophagus 3/10 of its length. Two large cells present at oesophago-intestinal junction (Fig. 1D).

*Male* (*n* = 21): Body length 11 579–25 592; maximum width 56–123. Whole oesophagus, 4855–9344 long; muscular oesophagus and stichosome, 210–374 and 4635–9015, respectively. Ratio anterior:posterior (the distance from the head to the beginning of intestine:the distance from the beginning of intestine to the tip of the tail), 1:1.2–1:2.5. Tail 14–38 long. Spicule 1437–2715 long (8–11% of body length) and slender (Fig. 1F and G); proximal end of spicule spatula-like (Fig. 1H). Spicular sheath non-spinous (Figs 1I, 1J, 2G and 2H). Posterior end of body broad and with 2 membranous lateral rays (Figs 1K and 2G). Two post-cloacal papillae present (Fig. 1K).

*Gravid female* (*n* = 20): Body length 16 532–31 485; maximum width 101–180. Whole oesophagus 5868–9249 long; muscular oesophagus and stichosome 148–436 and 5719–8581, respectively. Ratio anterior:posterior, 1:1.4–1:3. Tail, 12–25 long (Fig. 1C). Vulva with no elevated lips (Figs 1D and 2F) situated at 46–107 from oesophagus end; vagina directed posteriorly from vulva, and uterus containing 127–545 eggs. Eggs elongate with slightly protruding polar plugs (Fig. 1E) and uncleaved content, 60–71 long and 28–34 wide; egg wall thick with 2 layers: an inner hyaline layer and a thicker outer layer with fine longitudinal sculpture (Fig. 1E).

### Remarks

The morphology of specimens examined in this study using SEM expands upon the traditional description of *P. najae* provided by De (1998). SEM shows that the cephalic region displayed lips projecting out the oral opening (Fig. 2A). The body's midsection featured transverse striations on the ventral side (Fig. 2B and F). Unlike the 2 lateral bacillary bands described by De (1998) that extend between the nerve ring and cloaca, the SEM specimens examined in this study featured a dorso-laterally covered bacillary band extending from the middle of the body to the cloaca (Fig. 2F). This bacillary band was observed to contain scattered bacillary cells (Fig. 2C).

De (1998) examined a single host specimen without conducting any studies on the parasite–host relationship. In the present work, *P.* (*O*.) *najae* exhibited a significant degree of morphometric variation among the cobra specimens (see Table S1). For example, the longest average body length of the parasite (20 738–30 483 μm, *n* = 8) was observed in a cobra (sample no. SN017) with a 109.5 cm snout-vent length (SVL), while the smaller nematodes (11 579–17 167 μm, *n* = 8) were found in a larger cobra (SN007: 134.5 cm SVL). On the other hand, the largest snake (SN055: 147.8 cm SVL) has larger parasites compared to the smaller snake (SN013: 136.0 cm SVL) (see Table S1). The result suggests that no apparent relationship exists between host and parasite size. Other morphometric characteristics, including body width, oesophagus length, anterior part length and tail length, also displayed varying levels of variation with unclear patterns.

De's (1998) study showed no difference in body length between males and females. However, a difference was evident in all specimens from monocled cobras. Statistical analysis confirmed that female individuals typically exhibit a greater body length (*U* = 122.0, *P* = 0.022), width (*U* = 25.0, *P* ⩽ 0.001), tail length (*U* = 132.0, *P* = 0.042) and posterior length (*U* = 128.0, *P* = 0.032) compared with males (see also Table S3).

### Molecular characterization and phylogenetic position

**Figure 3.**
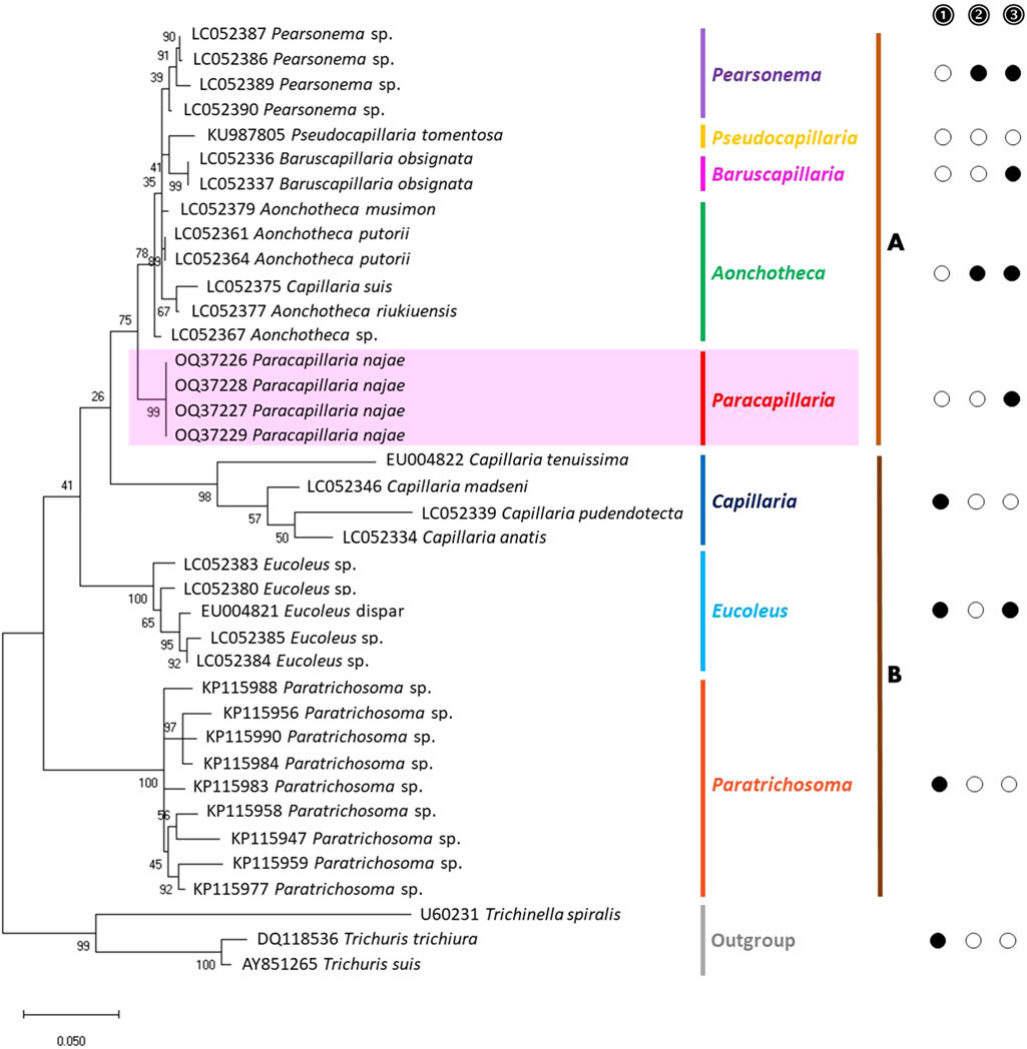
Phylogenetic analysis of capillariids based on 18S rRNA inferred by MEGAX using maximum likelihood (ML) method (branch length scale bar indicates number of substitutions per site). Coloured lines indicate capillariid genera genetic data obtained from GenBank. Red line indicates the genus of specimens = *Paracapillaria* used in the present study. The capillariids were divided into 2 clades; clade **A** is characterised by non-spinous specular sheath, and clade **B** is characterised by spinous specular sheath. 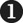 = spicular sheath of male spiny; 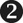 = caudal end of male with lateral alae; 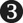 = tail of male with membranous bursa supported by 2 lateral rays; ⬤ = yes, ○ = no (synonym remark: *Capillaria suis* = *Aonchotheca suis*).

**Figure 4.**
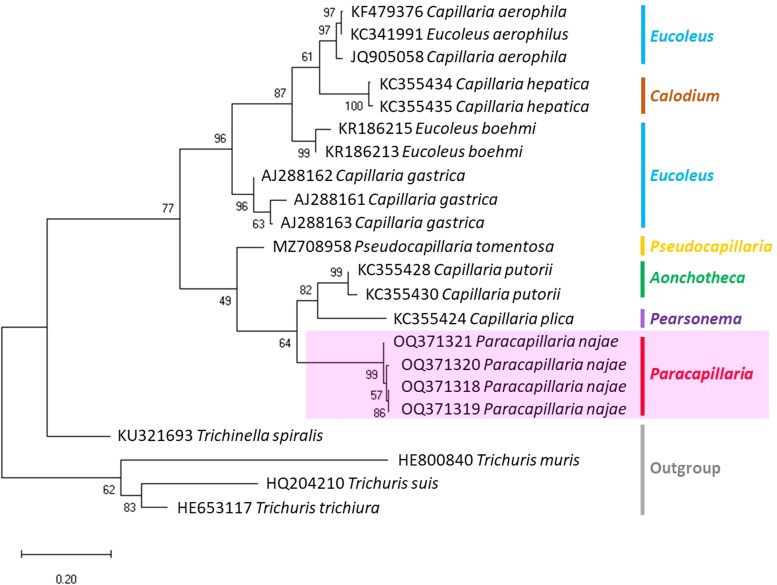
Phylogenetic analysis based on *COI* gene of capillariids inferred by MEGAX using ML method (branch length scale bar indicates number of substitutions per site). Coloured lines indicate capillariid genera genetic data obtained from GenBank. Red line indicates the genus of specimens = *Paracapillaria* used in the present study (synonym remark: *Capillaria hepatica* = *Calodium hepaticum*, *Capillaria gastrica* = *Eucoleus gastricus*, *Capillaria putorii* = *Aonchotheca putorii* and *Capillaria plica* = *Pearsonema plica*).

The molecular characterization of *P.* (*O.*) *najae* involved amplifying and sequencing the nuclear 18S rDNA and the mitochondrial *COI* genes, resulting in amplicon lengths of 800 and 288 bp, respectively. The phylogenetic analysis outcomes clearly revealed that *P. najae* is a distinct species within the taxonomic classification of Capillariidae. It was found to be closely related to capillariids such as *Aonchotheca*, *Baruscapillaria*, *Pseudocapillaria* and *Pearsomena*, which also possess a spicular sheath without spines (Figs 3 and 4). The genetic difference between *P. najae* and other capillariids ranged 2–14% (for 18S) and 16–26% (for *COI*), respectively. The closest genetic distances were observed between *P. najae* and members of *Aonchotheca*, 2–3% for 18S rRNA and 17–18% for *COI*.

## Discussion

The diagnostic morphology of the parasites discovered in the monocled cobra *N. kaouthia* in central Thailand aligns with that of *P.* (*O.*) *najae* by De (1998) in the Indian cobra *N. naja.* This includes the presence of a spatula-like spicule, a broad caudal end with 2 membranous lateral rays, a non-spinous spicular sheath with transverse striation in males, a vulva opening without elevated lips in females and other characteristics. These findings confirm the taxonomic identification of the examined specimens as *P. najae* (*sensu* De, 1998).

However, morphological discrepancies were found when comparing the descriptions examined by De (1998) to variations of *P. najae* observed among hosts which were not previously noted. The notable difference pertained to the presence of a dorso-lateral bacillary band with scattered bacillary cells extending from approximately 2/5 of the total body length, which contradicted De's (1998) findings of 2 lateral bacillary bands. This feature appears to be delicate, requiring examination beyond the capabilities of a light microscope. It is possible that De (1998) examined specimens of *P. najae* without using SEM or cross-section technique, which may have contributed to the lack of clarity regarding this particular character.

Additionally, several measurement-related characters, particularly body length and width, exhibited broader ranges in this present study compared to De's (1998) study, where only 1 host specimen was examined. The inclusion of a larger number of cobra host specimens in this study allowed for a more comprehensive range of measurements, enabling a more accurate assessment of morphological proportions and facilitating identification for the species.

Moreover, a high degree of morphological variation was observed among *P. najae* specimens found in different cobra hosts, with minimal overlap (Table S1). The underlying factors responsible for this variation remain unclear, and a more extensive sampling of both hosts and parasites is necessary to gain a deeper understanding of this pattern. Intraspecific variation, including a range of intrinsic characteristics and extrinsic factors (Thompson and Lymbery, 1990; Benesh and Kalbe, 2016), such as foraging abilities (Barber, 2005), duration of parasitic infection (Gems, 2000) or host body temperature (Benesh *et al*., 2021), may contribute to this variation.

Interestingly, a juvenile snake examined in the present work was infected with larger parasites on average than many adult snakes. While previous studies have reported a decrease in nematode length with host age/stage (Chylinski *et al*., 2009), contradicting findings were observed with *P. najae* specimens in the recent work on the monocled cobra. Further investigations are needed to determine the underlying causes of these variations. However, these results emphasized the importance of examining large sample sizes for the measurable traits used to identify the species accurately.

Sexual dimorphism in the morphometric measurements of *P. najae* was evident within the same snake hosts, with females being larger than males. This contradicted De's (1998) study, which reported little difference in body length between females and males. Sexual size dimorphism resulting from differences in fecundity (Poulin, 1997), food and metabolic expenditure (So *et al*., 2012) and/or larval life histories (Benesh and Valtonen, 2007) is known to be common in parasitic nematodes such as *Ascaris* spp. (Wang, 2021), *Enterobius* spp. (Hugot *et al*., 1996) and Oxyurid nematodes (Morand and Hugot, 1998).

In addition to morphological data, the genetic information derived from 18S rDNA and mt *COI* markers provided valuable insights into the systematics of *P.* (*O.*) *najae*. This analysis confirmed that the genetic distinctiveness of *P. najae* from other parasite species belongs to different genera. This molecular characterization represents the first of its kind for *P. najae.* However, further genetic sequences from other paracapillariid species, particularly closely related within the subgenus *Ophidiocapillaria*, are necessary to establish the species status and elucidate its evolutionary relationships. Currently, the available data in GenBank on *Paracapillaria* species are limited, with only data on *Paracapillaria philippinensis* and its 18S rDNA sequence. However, the genetic data for *P. philippinensis* are insufficient for conducting comprehensive phylogenetic analyses, primarily due to the 18S rDNA's short length sequence (170–235 bp), which is a slowly evolving nuclear gene (Mallatt *et al*., 2004).

Based on the phylogenetic trees constructed using both nuclear and mitochondrial genes in the present study, the evolution of capillariids can be divided into 2 clades. *Paracapillaria najae* was grouped in the first clade (clade A) along with species from the genus *Pearsomena*, *Pseudocapillaria*, *Baruscapillaria* and *Aonchotheca*, characterized by a specular sheath without spines. The second clade (clade B) includes species from the genera *Capillaria*, *Eucoleus* and *Paratrichosoma*, characterized by a specular sheath with spines. *Paracapillaria najae* and other capillariid members have diverged evolutionarily and infect a variety of vertebrate hosts (Moravec, 1986; Moravec and Justine, 2020).

While *P. najae* (revealed in the present study) and *P. sonsinoi* have the ability to infect multiple host species, most paracapillariid species display host specificity. This supports the notion of a coevolutionary relationship between host and parasite species (Buckingham and Ashby, 2022). On the other hand, the ability of *P. najae* to infect multiple hosts remains poorly studied, although such infections in other parasite species have been proposed to be influenced by parasite transmission patterns and virulence (Rigaud *et al*., 2010). *Paracapillaria najae* infects both *N. naja* and *N. kaouthia*, 2 cobra species obtained from geographically distant regions. It is plausible that the parasite is transmitted through shared prey consumed by both cobra species, such as rodents, chicks, amphibians or other reptiles (Cox *et al*., 2012; Kalki *et al*., 2022). To confirm the multi-host infection capability of *P. najae*, investigating other generalist snakes, such as rat snakes (*Ptyas* spp.) and the Siamese spitting cobra (*Naja siamensis*), which have similar prey preferences as the monocled and Indian cobras (Cox *et al*., 2012) is suggested.

In Oriental regions, there is a wide distribution of *P. najae* within overlapping host species. De (1998) collected a sample from the Indian cobra from West Bengal, India, while the infected monocled cobra was found in Bangkok, Thailand, a region more than 1700 km away. Nonetheless, the distribution of the monocled cobra overlaps with that of the Indian cobra in eastern India (Leviton *et al*., 2003; Cox *et al*., 2012; Captain and Whitaker, 2016). Therefore, the coevolutionary relationship between host and parasite likely reflects the parasite's existence in the same areas (Best *et al*., 2010; Rigaud *et al*., 2010; Ebert and Fields, 2020). *Paracapillaria sonsinoi*, another *Paracapillaria* species, also exhibits multiple hosts and a wide distribution, and its hosts include the green whip snake *Hierophis viridiflavus* in Italy (Skryabin *et al*., 1957), the diamondback water snake *Nerodia rhombifera* in the USA and the viperine water snake *Natrix maura* in France (Moravec, 1986). Given that monocled cobras exhibit population divergence between different geographical regions (Ratnarathorn *et al*., 2019, 2023; Shi *et al*., 2022), exploring whether the parasite demonstrates similar clustering patterns that reflect its hosts' divergence would be intriguing. The broad distribution of *P. najae* in Oriental regions and its association with multiple host species, including the Indian cobra and the monocled cobra, emphasizes the importance of understanding coevolutionary host–parasite relationships. Investigating whether the parasite displays the same clustering pattern as its hosts could provide valuable insights into the evolutionary history and ecology of *P. najae*.

However, it is important to acknowledge the limitation of this study, which is the relatively small host sample size. Increasing the sample size may improve the robustness and reliability of the conclusion within the context of the species and regions studied. Additionally, the lack of DNA sequence information for *P. najae* collected from the Indian cobra in West Bangor, India, hindered a more comprehensive genetic comparison.

In conclusion, the morphological redescription, illustrations and molecular characterization of *P. najae* support its species identification. The study extends our knowledge of *P. najae* infection and distribution in snake hosts, highlighting its potential as a multi-host parasite with different host species in Oriental regions. The variation in *P. najae* specimens observed across different snake hosts suggests the need for further research to unravel the complexities of its life cycle and host–parasite interactions. Understanding the coevolutionary relationship between hosts and parasites contributes to understanding snake distribution and the diversity of parasitic nematodes. Examining additional snake species and their prey, along with the genetic clustering patterns of *P. najae*, could provide valuable insights into the parasite's evolutionary history and ecology. This work provides valuable insights into the evolutionary and ecological processes shaping parasite diversity and distribution.

## Data Availability

The data that support the findings of this study are available from the first and corresponding authors upon reasonable request.

## Supplementary material

For supplementary material accompanying this paper visit https://doi.org/10.1017/S0031182023000707. click here to view supplementary material
